# Targeted Enrichment: Maximizing Orthologous Gene Comparisons across Deep Evolutionary Time

**DOI:** 10.1371/journal.pone.0067908

**Published:** 2013-07-02

**Authors:** Shannon M. Hedtke, Matthew J. Morgan, David C. Cannatella, David M. Hillis

**Affiliations:** 1 Department of Integrative Biology and Center for Computational Biology and Bioinformatics, University of Texas at Austin, Austin, Texas, United States of America; 2 Texas Natural Science Center, University of Texas at Austin, Austin, Texas, United States of America; State Natural History Museum, Germany

## Abstract

Estimated phylogenies of evolutionarily diverse taxa will be well supported and more likely to be historically accurate when the analysis contains large amounts of data–many genes sequenced across many taxa. Inferring such phylogenies for non-model organisms is challenging given limited resources for whole-genome sequencing. We take advantage of genomic data from a single species to test the limits of hybridization-based enrichment of hundreds of exons across frog species that diverged up to 250 million years ago. Enrichment success for a given species depends greatly on the divergence time between it and the reference species, and the resulting alignment contains a significant proportion of missing data. However, our alignment generates a well-supported phylogeny of frogs, suggesting that this technique is a practical solution towards resolving relationships across deep evolutionary time.

## Introduction

Biologists have made great strides in reconstructing the evolutionary history of Life. Numerous studies have demonstrated the importance of sampling a sufficient number of both genes and taxa across a given phylogeny to produce an accurate estimate of evolutionary history [1], [2], [3], [4], [5], [6]. The number of available orthologous sequences for many taxa has greatly increased by comparing whole genomes [7], [8], [9], [10] or expressed sequenced tags (ESTs; e.g., [11], [12], [13], [14]). Although these two approaches have proven useful, generating the initial sequence data typically requires high-quality tissue samples and considerable effort for each species. Thus, there is often a trade-off between studies that compare and analyze many genes and those that thoroughly sample among taxa [15].

Next-generation sequencing (NGS) enables unprecedented access to large DNA data sets. Although comparisons of whole genomes produce the largest set of orthologs, whole genome studies are not yet routine–or economically feasible–for the majority of the >1.7 million species of life. Even when available, large portions of the genome evolve too rapidly to be aligned reliably even among closely related species, so only relatively small regions of coding sequences are useful in phylogenetic analyses. Enrichment refers, broadly, to increasing the representation of particular regions of the genome prior to sequencing. This reduces the overall number of NGS reads necessary to sequence the regions of interest, and thus the overall cost of the project. One such approach is sequencing the transcriptome, rather than the whole genome. Messenger RNA is extracted from fresh tissue and used to produce cDNA libraries, which are sequenced and used to build phylogenies (as in [16], [17], [18]). These data sets are enriched for expressed genes, and contain only exon sequences. Although useful for phylogenetic analyses, transcriptome sequencing requires mRNA from fresh tissues, and cannot be used with genetic resource collections, museum specimens, or tissues that have been stored in ethanol.

An emerging technique for generating large collections of orthologous genes is to enrich genomic DNA for target regions prior to sequencing. Oligonucleotide probes for the target regions are designed from known sequences and fixed to microarrays or placed in solution, and hybridized to genomic DNA. Only DNA that successfully hybridizes to these probes is sequenced, and thus the resulting sequences are enriched for these target regions [19], [20], [21], [22]. For broad-scale phylogenetic analysis, the major advantage of targeted enrichment over sequencing transcriptomes is the ability to use preserved and frozen tissue collections.

We explored the usefulness of targeted enrichment for investigations of diverse, non-model organisms separated by deep divergences–taxa crucial for joining the major branches of the Tree of Life. The majority of studies have used enrichment to target and sequence exons for multiple individuals of the same species (also called “exon capture” or “re-sequencing” [20]). Recent work seeks to expand the application of targeted enrichment to species for which no genome yet exists. Enrichment probes have been designed from sequenced genomes [23], [24], [25], [26] and transcriptomes [27]. These experiments have compared enrichment among species that share a relatively recent common ancestor (less than ∼65 mya; [23], [24], [25], [27], [28]) or among very distantly-related species (over ∼100 mya; [25], [26]). We wished to examine enrichment success across a wider range of divergence times, using a single reference genome to identify target regions for use in phylogenetic analyses.

**Figure 1:**
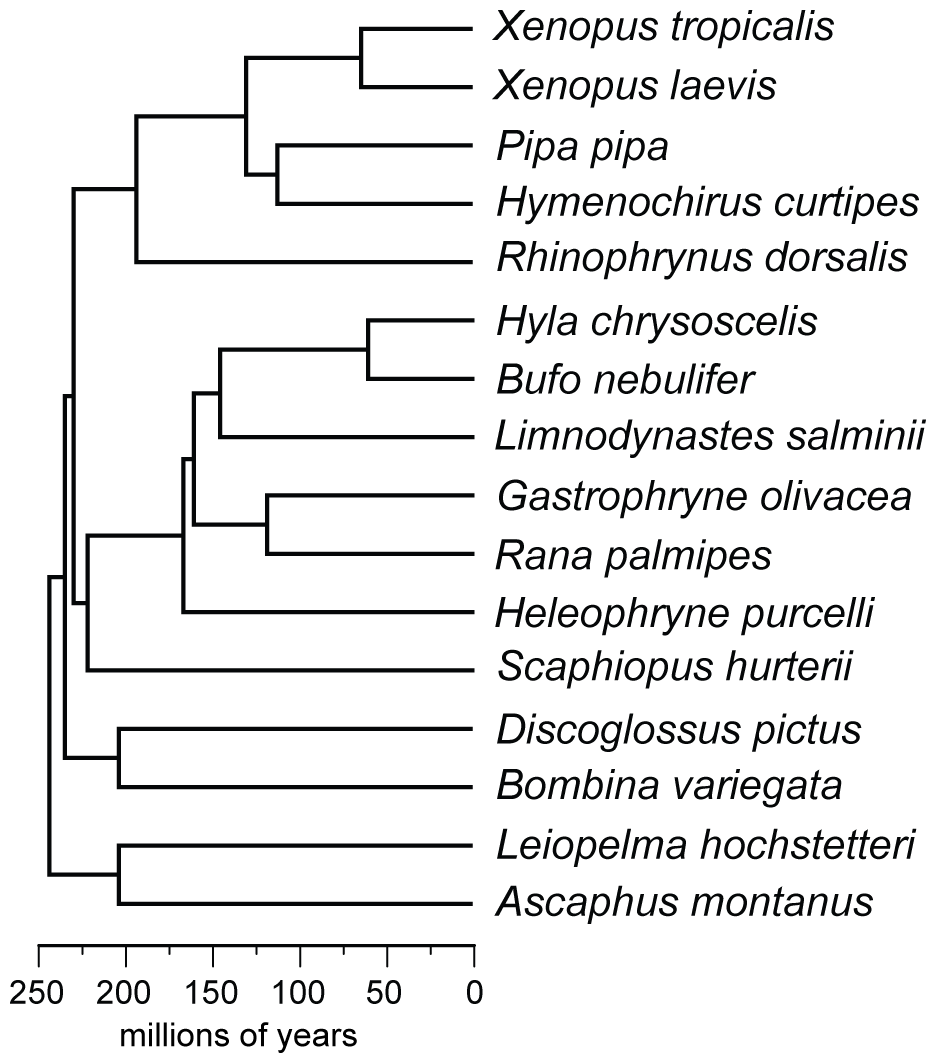
Divergence times among 16 species of frogs. Estimates from Evans et al. [54] and Bossuyt and Roelants [56].

We investigated the potential of targeted enrichment to capture and sequence exons across frogs, an ancient, diverse clade with limited existing genomic resources. This group comprises at least 6285 extant species [29] that span an evolutionary divergence of approximately 250 million years. The only frog genome available at the start of our study was that of the Western Clawed Frog, *Xenopus* (subgenus *Silurana*) *tropicalis* [30]. We used this draft genome as the reference to design probes and enriched targeted exons from 16 species from across the diversity of frogs. Neobatrachia contains >95% of frog species, and the most recent common ancestor between neobatrachians and *Xenopus* is dated at >200 million years ago (Figure 1). We show that although both solution-based and array-based techniques for DNA enrichment are successful, the solution-based method appears more effective and practical, particularly between organisms that share a common ancestor between ∼65 and 200 million years ago. We find that this approach can be used to compare and analyze a broad range of orthologous sequences from genetic resource collections to produce well-supported phylogenetic estimates of deeply diverged lineages.

## Materials and Methods

### Sample Preparations

Frozen or ethanol-preserved liver and muscle tissue samples were loaned with permission from museum collections for 16 frog species (Table 1). DNA was extracted using a standard phenol-chloroform procedure; approximately 0.01–0.04 g of tissue was used for each extraction. The Genomic Sequencing and Analysis Facility at the University of Texas at Austin created 454 or SOLiD libraries for each species according to the published protocols for two different techniques [31], [32]; these library protocols require 3 µg of DNA extract for each sample.

**Table 1:** Collection data for tissue samples.

Species	Collection Number
*Ascaphus montanus*	MVZ 187733 (TMT 107)
*Bombina variegata*	TMT111 (from Jacek Szymura)
*Bufo (Incilius) nebulifer*	TNHC 62000 (DCC 3107)
*Discoglossus pictus*	TNHC-GDC 1150 (DMH 88–214)
*Gastrophryne olivacea*	TNHC 65364
*Heleophryne purcelli*	TNHC 85525
*Hyla chrysoscelis*	TNHC 64115 (DCC 3883)
*Hymenochirus curtipes*	TNHC 77214
*Leiopelma hochstetteri*	NMNS 29595-5 (DMG 2229)
*Limnodynastes salmini*	TNHC 61075 (DCC 2898)
*Pipa pipa*	TNHC 77277
*Rana (Lithobates) palmipes*	AMNH A118801 (CWM 18089)
*Rhinophrynus dorsalis*	LACM 122913 (CSL 6208)
*Scaphiopus hurterii*	TNHC 85075 (DCC 3006)
*Xenopus laevis*	TNHC 77309
*Xenopus (Silurana) tropicalis*	TNHC 77280

AMNH = American Museum of Natural History, New York; CSL = Carl S. Lieb; CWM = Charles W. Myers; DCC = David C. Cannatella; DMG = David M. Green; DMH = David M. Hillis; LACM = Los Angeles County Museum; MVZ = Museum of Vertebrate Zoology, University of California, Berkeley; NMNS = National Museum of Natural Science, Ottawa; TMT = Ted M. Townsend catalog; TNHC = Texas Natural History Collection (Voucher), University of Texas, Austin; TNHC-GDC = Genetic Diversity Collection of the Texas Natural History Collection.

### Target Sequence Selection

To generate targets for probe design, independent of enrichment protocol used, nuclear protein-coding sequences (CDS) longer than 300 bp were extracted from the *X. tropicalis* reference genome v.4.1 [30] using the annotations in Xentr4_FilteredModels1.gff (http://genome.jgi-psf.org/Xentr4/Xentr4.download.ftp.html). Custom perl scripts were used to generate all possible 120-mer sequences (“candidate baits”) from these sequences with a one-nucleotide offset. Candidate baits were assessed for target specificity to the reference genome by comparing each bait against the entire *X. tropicalis* genome using BLASTN [33]. This was an *in silico* test of whether each candidate bait was likely to perform well *in vitro.* A well-designed probe would hybridize only to the coding sequence of interest (the “target exon”), and thus could be successful at enriching the DNA sample for that exon prior to sequencing. We rejected candidate baits that hit multiple regions of the reference genome (with a bitscore >36), because that bait sequence would likely perform poorly in enrichment. We further screened candidate baits for GC content; only candidate baits with a GC content of 40–60% were retained. We assembled the remaining baits into contigs and only retained baits that formed contigs >300 bp long. These selection filters resulted in a pool of 2,823 candidate exon sequences. Finally, target sequences were selected such that the total number of target nucleotides was ∼450 kb to minimize the costs of obtaining adequate sequencing depth and to include a range of bait densities (bait tiling offset ∼20–100 bp). This final set of target sequences comprised 933 contigs across 876 unique exons for a total of 458,463 bp.

### Target Enrichment

We tested two techniques for enrichment: an array-based enrichment followed by 454 sequencing (Roche NimbleGen, Inc.), and a solution-based enrichment followed by SOLiD sequencing (Life Technologies Corp.). For the array-hybridization approach, the *X. tropicalis* genomic scaffold locations for each target sequence were submitted to Nimblegen for probe design and array manufacture (Roche Nimblegen, Inc.). For the solution hybridization approach, 7,068 bait sequences were selected across the 933 target loci and submitted to Agilent for SureSelect Target Enrichment solution manufacture (Agilent Technologies, Inc.).

For Nimblegen's array-hybridization, “*C_0_t*” DNA is required to block non-specific hybridization [31]. *C_0_t*-1 DNA is enriched for multi-copy sequences (the name is derived from the procedure used to produce the enriched DNA). When it is used in enrichment protocols, *C_0_t*-1 DNA is intended to bind to similar sequences in the sample, such as DNA sequences for ribosomal genes, thereby reducing non-specific binding to the targets on the array. We used a 3∶1 mixture of commercial human *C_0_t*-1 DNA and frog-derived *C_0_t*-1 DNA. Frog-derived *C_0_t*-1 DNA was produced using the protocol developed by Zwick et al. [34]. We tested enrichment success in *Pipa pipa* using both *X. tropicalis*-derived and *P. pipa*-derived *C_0_t*-1 DNA.

The enrichment protocol for each technique was followed per manufacturer's instructions [31], [32], except that less than 500 ng of library was used for solution-based enrichment of three species, since the libraries were of low concentration (*Hymenochirus curtipes, Heleophryne purcelli, Xenopus laevis*). Hybridized libraries were sequenced using the 454 Titanium (Roche Nimblegen, Inc.) or SOLiD 3.0 plus (50- and 35-bp paired-end run; Life Technologies Corp.) next-generation sequencing technologies by the Genome Sequencing and Analysis Facility at the University of Texas at Austin. Reads have been deposited into the NCBI SRA database under project SRA051431 (http://www.ncbi.nlm.nih.gov/Traces/sra/).

### Analysis of Reads

To compare the array-based and solution-based techniques, we mapped the reads to the reference genome using SHRiMP v.2.0.2 [35]. The majority of species were enriched using the solution-based protocol (see Results) and reads mapped to the reference genome using BFAST v.0.6.5a [36]. We attempted *de novo* assembly of reads using Velvet [37], one of the only assemblers designed to assemble reads in color space (rather than nucleotide base pairs). The few contigs formed did not blast to the reference genome, suggesting errors in assembly. When enrichment is successful, hundreds of reads can be generated from the same region, and Velvet may have interpreted these as repetitive elements.

Prior to building contigs, we removed possible PCR duplicates and discarded regions without sufficient sequencing depth to counter sequencing error (5× depth for 454 data; 15× for SOLiD) using a custom perl pipeline incorporating SAMtools [38]. Contigs varied in uniformity (the proportion of a target sequenced). For each species, if a target exon that was sequenced had an (arbitrarily-selected) uniformity of less than 40%, we removed it from the dataset. We combined the remaining contigs into an aligned matrix based on their mapped position relative to the reference genome. Target exons in the matrix that contained data from only one species were removed from the alignment. Because sequencing depth also varied within exons, columns in the matrix that contained sequence from only one species were also filtered out, yielding the final matrix (deposited into TreeBASE: http://purl.org/phylo/treebase/phylows/study/TB2:S14191). A second filtering step was performed to remove all columns with *any* missing data. We thus analyzed two matrices: a large matrix with missing data (74%), and a much smaller matrix with no missing data.

### Simulations

Since we built contigs from our reads using a mapping program (BFAST [36]), we wished to explore whether this would introduce error when mapping divergent species. A particular set of reads containing highly divergent sequences may have been enriched (hybridized to probes and sequenced) but not mapped. Thus, our method of evaluating enrichment may underestimate success. To explore whether our method of evaluating enrichment would distinguish between failed and successful experiments, we used a simulation approach. Since simulated reads are generated under known conditions, they can be used to evaluate the accuracy of our pipeline under those conditions. We simulated datasets of 10^7^ SOLiD paired reads (2×10^7^ total) from target sequences using dwgsim utility in the dnaa package v. 0.1.2 (http://sourceforge.net/apps/mediawiki/dnaa/). This package simulates reads from a reference genome given the average percent divergence expected between the read and the reference, while incorporating standard SOLiD error rates into the pool of reads for each pairwise distance. We simulated reads based on our target regions, across five average pairwise distances spanning the expected range of divergences for orthologous, nuclear protein-coding genes across Anura (0, 0.06, 0.12, 0.18, 0.24). We then mapped these simulated reads back to the reference genome using BFAST [36] and evaluated whether the simulated reads mapped to the correct target regions. If there is an effect of divergence on our ability to detect enrichment, then we would expect the number of reads mapped correctly to decrease as the pairwise distance increases (i.e., an increase in false negatives). Divergence could also lead to inaccurate mapping, in which case the number of reads mapped incorrectly would also increase as the pairwise distance increases (i.e., an increase in false positives).

### Assessing Variation in Enrichment among Exons

Our expectation was that the more evolutionarily conserved an exon, the more likely it is to successfully enrich across all frogs. However, because we lack genomic information for most species in our study, we cannot calculate relative rates of evolution of enriched exons compared to exons that fail to enrich. Thus, as a rough proxy measure for how conserved exons might be, we used the p-distance (uncorrected sequence divergence) between the reference sequence for an exon and the sequence from the species with the highest number of enriched exons, *X. laevis*. We used the p-distance rather than a distance that corrects for unobserved substitutions because we were interested in the actual proportions of differences between the two sequences, rather than the estimated number of substitutions that produced those differences. This measure is expected to better reflect the relative hybridization intensity between sequences. We compared the mean of these p-distances for enriched and un-enriched regions, both among exons and among subregions of exons only partially sequenced in some taxa. If the means and ranges of enriched versus unenriched regions overlap, then stochastic forces related to experimental variation may have driven the observed differences in enrichment success across exons. Alternatively, enriched regions could have a smaller mean p-distance between *X. laevis* and *X. tropicalis* than unenriched regions, suggesting that enriched regions are more conserved across the history of frogs.

### Phylogenetic Reconstruction

We used RAx-ML v. 7.3.0 [39] to analyze the final matrices, with 1000 bootstrap replicates to evaluate support for the tree. We performed both unpartitioned analyses and analyses partitioned by gene using the GTRCAT approximation of sequence evolution. We analyzed both the entire matrix and the matrix that had columns with missing data removed to examine whether the completeness of the alignment affected topology. Finally, we performed a test for the best-fitting model of evolution on each target exon sequenced in more than four species by scoring a neighbor-joining tree in PAUP* v. 4b10 [40] and analyzing the results using Modeltest v. 3.7 [41] under the Akaike Information Criterion [42]. The maximum-likelihood topology and bootstrap proportions (from 100 bootstrap replicates) were determined for each of these target exons using Garli v. 2.0 [43]. PAUP* [40] was used to compare bootstrap proportions across individual gene trees. We additionally ran maximum-likelihood analyses using a concatenated alignment excluding exons that failed to infer *Rhinophrynus dorsalis* as sister to the pipid frogs.

For each exon sampled in all 13 taxa (n = 23), we determined the posterior distribution of topologies using MrBayes v.3.2.1 [44] and the best-fit model of sequence evolution with two runs with four chains for 5 million generations, sampling every 500 generations. Stationarity was assessed using Tracer v.1.5 [45], and convergence when the topological standard deviation between runs calculated by MrBayes was under 0.01. All twenty-three exons apparently reached stationarity and convergence rapidly, and the first 10% of each run was discarded as burn-in. The posterior distribution of tree topologies was used to calculate sample-wide Bayesian concordance factors for each bipartition using BUCKy v.1.4.2 [46].

## Results

### Simulations

**Figure 2:**
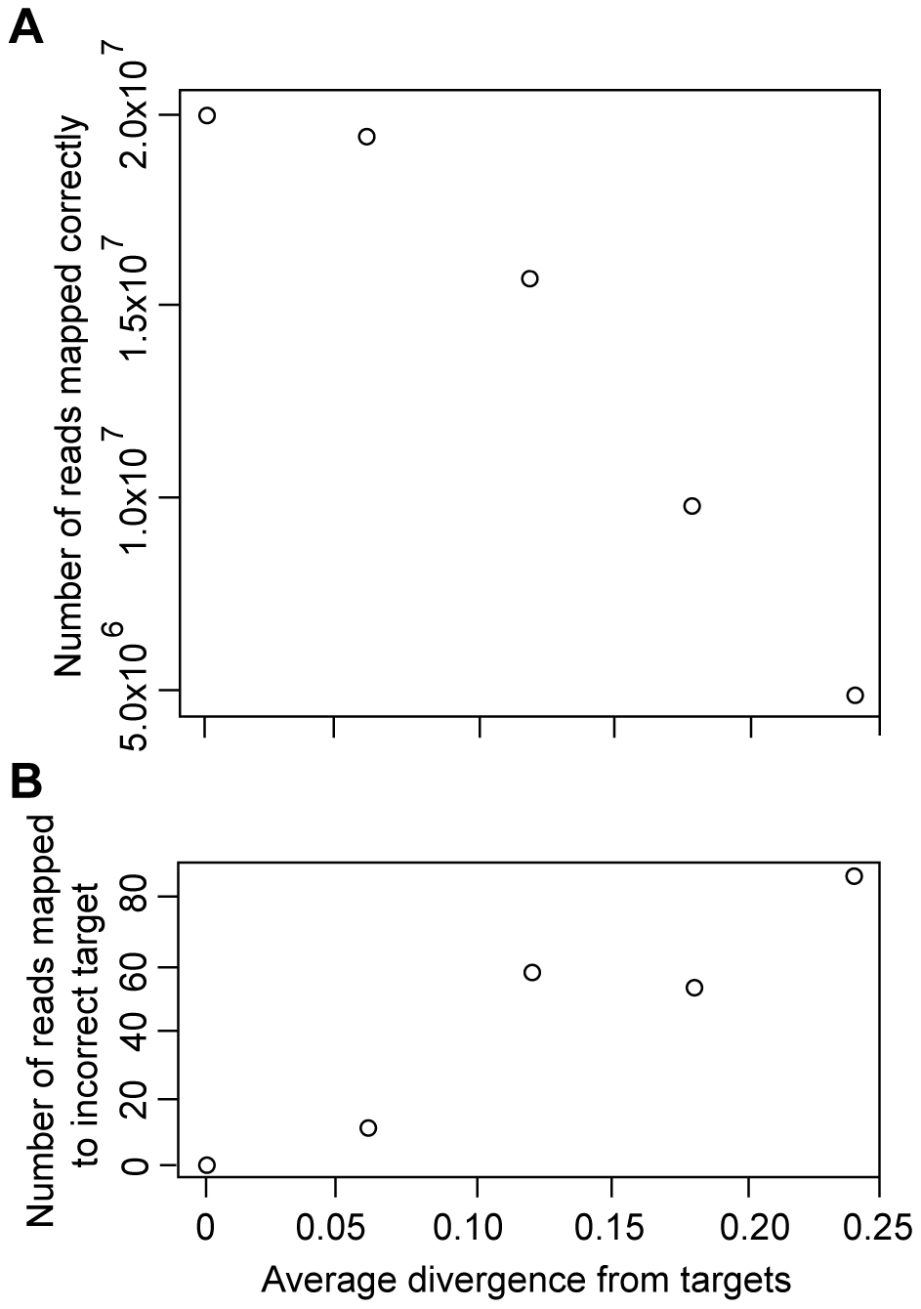
Mapping success of simulated reads. Reads simulated with average expected differences between 0 and 24% from target exons on the reference sequence, mapped against the entire *Xenopus tropicalis* genome. A) Number of simulated reads (out of 2×10^7^) mapped to the correct target exon in the genome. B) Number of simulated reads mapped to the incorrect target exon on the reference genome.

Simulations were used to assess whether values for enrichment were affected by our analysis of sequence reads. The degree of pairwise differentiation between a particular species and *Xenopus tropicalis* might affect hybridization of interspecific DNA to the probe sequences, or it might affect successful mapping of reads to the reference sequence, or it might affect both of these steps. Our simulations assumed that hybridization was successful, and that the pool of DNA sequenced only contained DNA from target exons. We found that reads simulated on the target regions only very rarely mapped to the wrong target exon, even when the average p-distance between the reference and simulated reads was as high as 24%. Thus, mapping was conservative in that it did not overestimate enrichment. However, as divergence from the reference sequence increased, the number of reads that failed to map to their correct target exon increased (Figure 2), and thus it is possible that we underestimated enrichment.

### Enrichment

Enrichment is measured as the x-fold increase in sequencing of target regions over the expectation from random (shotgun) sequencing. This expectation is based on the assumption that a region is likely to be sequenced in proportion to its representation in the genome. The purpose of enrichment is to increase the representation of target regions in the pool of DNA. Thus, we calculated enrichment as the percent of basepairs that mapped to target exons at >5× coverage (454 sequences) or >15× coverage (SOLiD sequences) divided by the percent of basepairs in the genome that is target sequence (as in [19]), assuming a genome size of 1.7 Gbp [30]. By this measure, enrichment was successful for both the array-based and the solution-based technique (Table 2). We found that the array-based technique required species-specific *C_0_t*-1 DNA: when we used *Xenopus C_0_t*-1 DNA in the experiment to enrich *Pipa pipa*, enrichment failed, but enriched 585-fold when *Pipa C_0_t*-1 DNA was used in the experiment (Table 2). The solution-based protocol did not require *C_0_t*-1 DNA for successful enrichment, and so we used this protocol to generate our data matrix for phylogenetic analysis.

**Table 2:** Enrichment in *Xenopus tropicalis* and *Pipa pipa* for 933 target regions of the *Xenopus tropicalis* genome after array-based (454 sequencing) and solution-based (SOLiD, 35-bp sequencing) protocols.

	Array-based (5× minimum depth)			Solution-based (15× minimum depth)	
	*X. tropicalis*	*P. pipa* (*Xenopus C_0_t*)	*P. pipa* (*Pipa C_0_t*)	*X. tropicalis*	*P. pipa*
Number of reads	251766	66017	195676	21575543	49058284
Number of reads that map to genome uniquely	125755	350	14540	12150987	4724097
Number of reads that map to targets uniquely	11449	0	13237	10295604	3623549
Number of targets	681	0	180	768	606
Enrichment	1777	0	585	2931	1621
Average uniformity per target	0.65	0	0.81	0.98	0.68
Number of targets with uniformity >50%	506	0	153	756	524
Number of bp that hit targets	219735	0	72373	362491	200489
Number of bp in targets with uniformity >50%	236099	0	98914	541324	210410

We assessed capture, sequencing, and assembly efficiency of the solution-based protocol with our data for *Xenopus tropicalis*, the species used to design the hybridization probes. Using a strict criterion for successful enrichment (≥15× sequencing depth for at least 40% of basepairs in each exon), we successfully captured, sequenced and assembled data for 779 of 933 target regions (83.5%) in 756 unique exons. Of these 779 target regions, the mean percentage of basepairs successfully sequenced in each target was 99.44%, with 748 target regions (96.0%) being completely sequenced. In total, we sequenced 561,776 bp, of which 374,812 bp were from target regions and 186,964 bp were from flanking regions. Mean sequence identity between our contigs and the reference genome was 99.9% (97.3%–100%); 612 (78.6%) sequenced target regions were identical to the reference, whereas 12 (1.5%) contained internal gaps or missing data.

Across the diversity of frog species, enrichment ranged from a 33- to 2948-fold increase in target sequence compared to expectations from random sequencing (Table 3). For three species, the library was of lower concentration than required by protocol (*Hymenochirus curtipes, Heleophryne purcelli, Xenopus laevis*). Enrichment occurred in these experiments, but may not have been as successful as in those species for which protocol was followed precisely. For four species, the solution containing the RNA probes had been de-thawed more than twice prior, and these enrichments failed (*Rana palmipes*, *Gastrophryne olivacea, Limnodynastes salminii*) or had vastly reduced enrichment compared to earlier hybridization with fresh probes (*Pipa pipa:* first experiment, 1531-fold enrichment, second experiment, 580-fold enrichment). Because of the relative lack of success of *P. pipa*, these four reactions most likely failed because of experimental error and not because of shared rapid rates of genetic evolution in the three closely-related neobatrachians. For the 13 species for which capture was successful, the number of target exons sequenced ranged from 58–756 (after the conservative procedure of excluding possible PCR duplicates; see Methods; Table 3).

**Table 3:** Enrichment success for 16 species of frogs across 933 target exons designed from the *Xenopus tropicalis* draft genome.

Species	Number of read pairs	Number of targets hit	Enrichment	Average uniformity per target	Number of targets, uniformity >0.4	Number of bp, uniformity >0.4
*Xenopus tropicalis*	36922537	766	2948	0.987	756	564517
*Xenopus laevis*	48693088	739	2750	0.954	712	506266
*Hymenochirus curtipes*	42242254	573	1573	0.708	296	151769
*Pipa pipa*	45059526	400	761	0.489	105	49491
*Rhinophrynus dorsalis*	43267014	758	1978	0.667	292	153190
*Hyla chrysoscelis*	41786281	265	491	0.465	89	47885
*Bufo nebulifer*	43083447	753	1560	0.520	58	34325
Limnodynastes salminii*	47137178	164	220	0.334	32	17202
Rana palmipes*	41391719	83	102	0.297	2	885
Gastrophryne olivacea*	42246979	30	33	0.269	2	1208
*Heleophryne purcelli*	46746319	238	350	0.375	55	30734
*Scaphiopus hurterii*	41880799	360	770	0.548	159	75357
*Bombina variegata*	34098746	364	763	0.531	134	72285
*Discoglossus pictus*	50254177	411	913	0.573	184	95756
*Leiopelma hochstetteri*	46283396	404	698	0.434	73	40887
*Ascaphus montanus*	54250511	284	461	0.408	90	45056

Paired-end reads (50- and 35-bp) sequenced using SOLiD next-generation sequencing technology were mapped to the reference genome to assess enrichment at 15× depth. * denotes species not used in final phylogenetic analyses due to poor enrichment.

**Figure 3:**
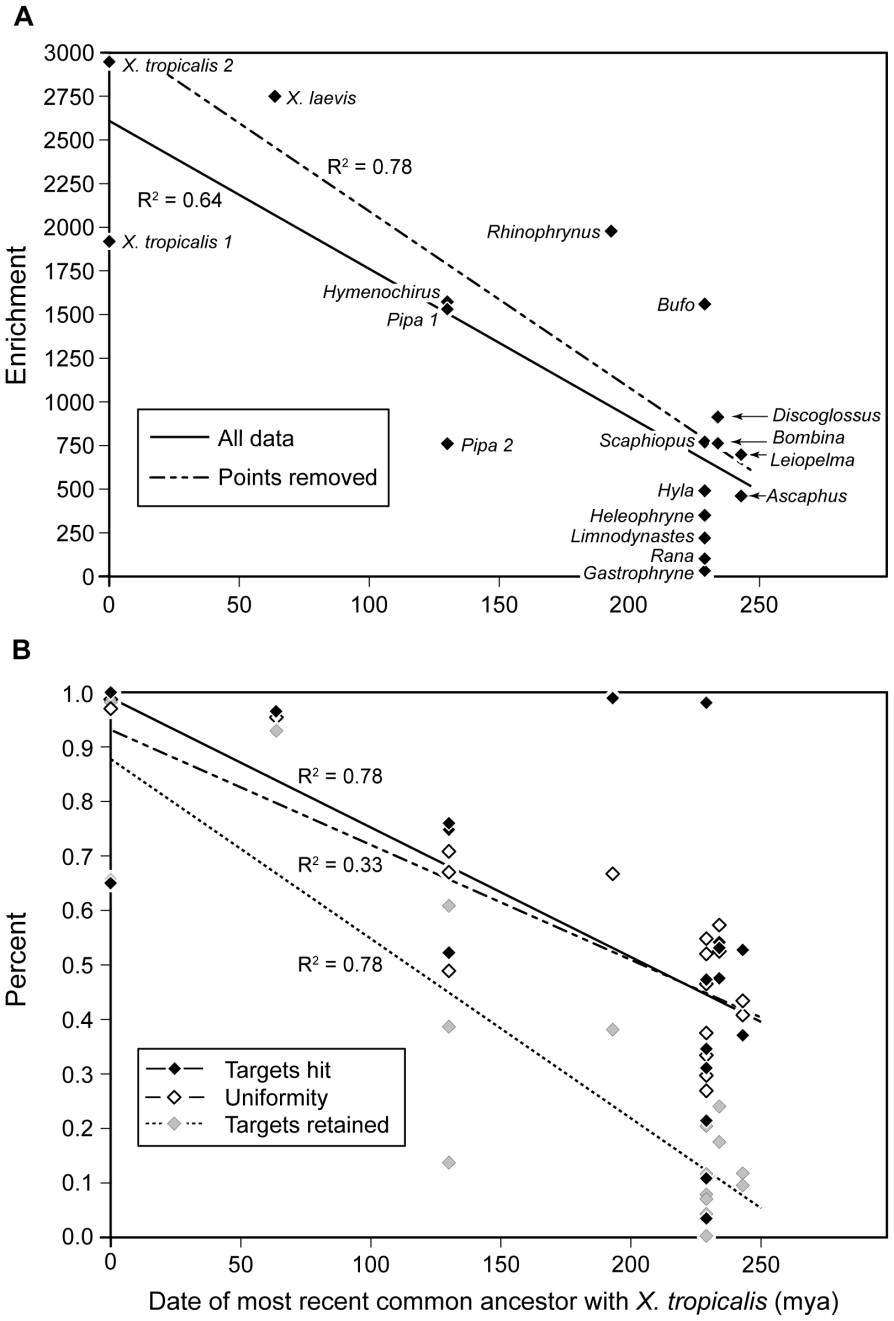
Effect of divergence time on enrichment success across 16 species of frogs. A) Enrichment (x-fold improvement in sequencing compared to random shot-gun sequencing). Paired reads (35- and 50-bp) were sequenced for all 16 species. *Xenopus tropicalis* and *Pipa pipa* were also independently sequenced for single 35-bp reads. Trend lines were computed for all data points, and after removing three data points that likely had reduced enrichment because of experimental error: *P. pipa* (paired run), *Rana palmipes*, and *Gastrophryne olivacea*. B) The number of targets for which any reads mapped for each species, the average uniformity for these targets (the proportion of basepairs sequenced at 15×), and the number of targets with a uniformity of at least 0.4.

**Figure 4:**
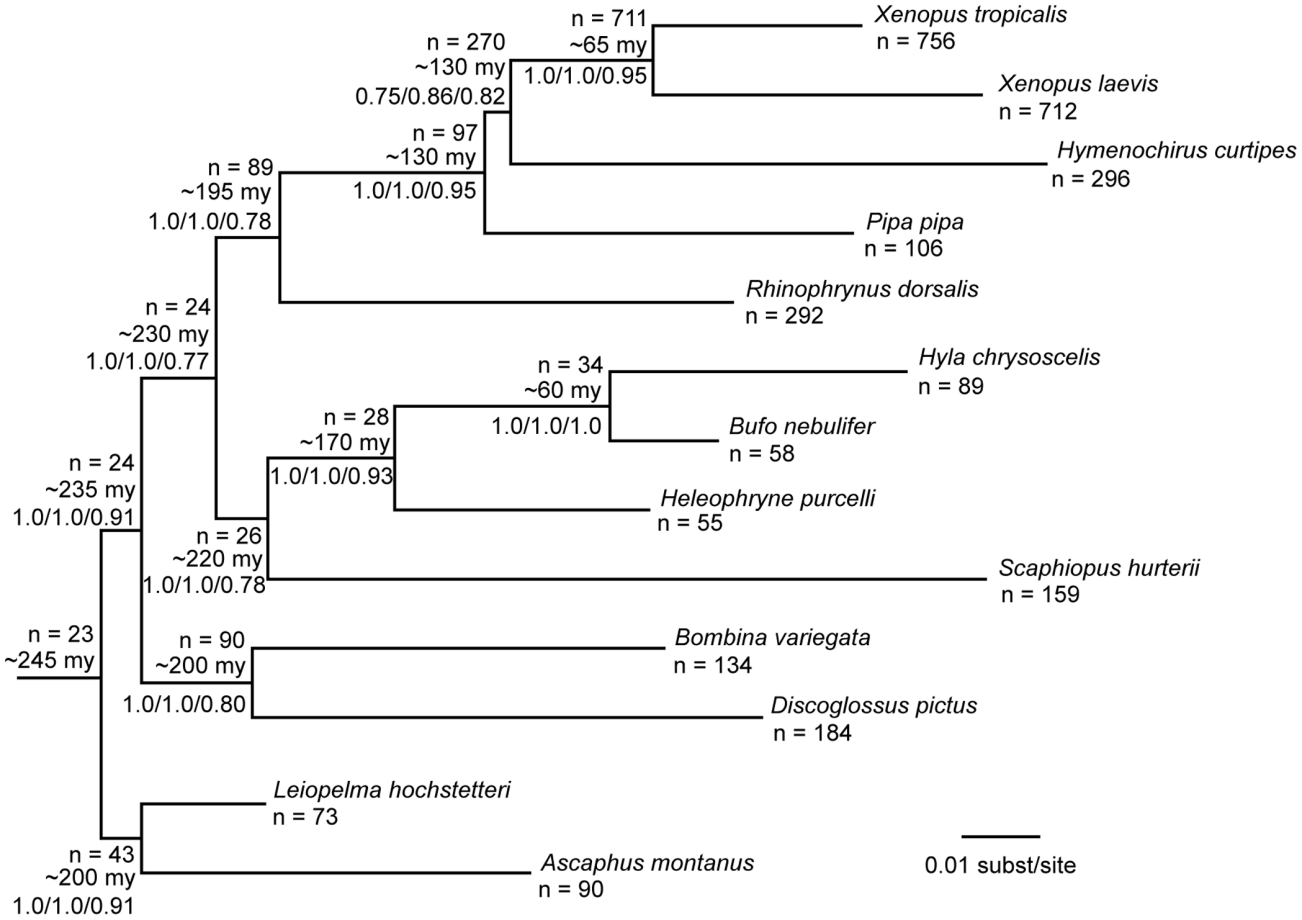
Maximum likelihood estimate of relationships among frog species based on 764 target exons. At each terminal taxon and internal node, we note the number of target exons sequenced for all species within the clade (n), the estimated time of divergence at each internal node, rounded to the nearest 5 my [54], [56], and support values: bootstrap proportion based on concatenation of all exons/bootstrap proportion based on a matrix with no empty cells/Bayesian concordance factor based on 23 exons enriched in all taxa.

As divergence time from the reference sequence increases, enrichment (and thus the number of target exons sequenced) decreases (Figure 3). Differences in rates of molecular evolution across genes may explain enrichment failure of one target compared to another because targets with greater sequence divergence will have reduced hybridization success. To test this hypothesis, we assumed that average rates of evolution of protein-coding genes do not differ across anurans, but rates of evolution do differ among genes. Under this assumption, we used the pairwise distance between *Xenopus tropicalis* and *X. laevis* as a measure of relative gene conservation. Exons that were successfully enriched in a species had a lower p-distance between *X. tropicalis* and *X. laevis* than those that failed (Table 4), suggesting greater molecular divergence results in lower enrichment success of an exon. This pattern was also observed within exons: regions of exons that were successfully sequenced in a species also had a lower p-distance between *X. tropicalis* and *X. laevis* compared to the interspecific distance for the entire exon, and thus were more likely to be slower-evolving regions. However, enrichment success across exons was not always hierarchical as might be expected if all these assumptions were true: for example, *Ascaphus montanus* and *Leiopelma hochstetteri* diverged from *X. tropicalis* at the same time in evolutionary history, yet have different numbers of exons enriched (90 and 73, respectively), and only 43 of these enriched exons are shared by both taxa (Figure 4).

**Table 4:** Mean percent sequence divergence between successfully enriched targets and reference sequence used to develop enrichment probes.

Species	Mean distance between referenceand sequenced exons	Mean distance between reference and *X.laevis* : enriched exons	Mean distance between reference and *X.laevis* : unenriched exons
*Xenopus tropicalis*	1.00 (0.02)	n/a	n/a
*Xenopus laevis*	5.65 (0.14)	n/a	n/a
*Hymenochirus curtipes*	7.59 (0.11)	4.17 (0.10)	6.55 (0.11)
*Pipa pipa*	5.69 (0.09)	3.25 (0.08)	6.07 (0.19)
*Rhinophrynus dorsalis*	8.55 (0.11)	4.32 (0.12)	6.45 (0.10)
*Scaphiopus hurterii*	9.89 (0.11)	4.12 (0.11)	6.09 (0.10)
*Heleophryne purcelli*	7.26 (0.11)	3.33 (0.10)	5.85 (0.09)
*Bufo nebulifer*	6.01 (0.11)	2.93 (0.09)	5.80 (0.09)
*Hyla chrysoscelis*	9.23 (0.11)	3.87 (0.12)	5.91 (0.09)
*Discoglossus pictus*	10.15 (0.11)	4.02 (0.10)	6.21 (0.10)
*Bombina variegata*	9.29 (0.12)	3.64 (0.10)	6.02 (0.09)
*Leiopelma hochstetteri*	6.08 (0.10)	3.34 (0.10)	5.83 (0.09)
*Ascaphus montanus*	8.84 (0.10)	4.06 (0.12)	5.88 (0.09)

Target exon sequences were based on the *Xenopus tropicalis* reference genome. Species were not enriched for all exons. To estimate whether more conserved exons were more likely to be enriched, distance calculations between *X. tropicalis* and *X. laevis* were made for each target exon enriched for a given species and for each target exon that failed to enrich for that species. Standard errors are given in parentheses.

### Inferred Phylogenies

Our complete matrix contained sequences for 727 exons for 13 taxa. Our inferred phylogeny (Figure 4) is congruent with other published phylogenies of frogs [47], [48], [49], [50]. Both the complete concatenated data set (13 species, 497,239 bp, 74% missing) as well as the data set with no missing data (13 species, 8,494 bp, 0% missing) generated the same maximum-likelihood topology, regardless of partitioning scheme. This topology was also identical to the Bayesian concordance tree based on those 23 exons sampled across all species. The number of exons shared among all taxa in a clade was highest within the genus *Xenopus*, with 711 exons (comprising 729 individual target regions) sequenced in both *X. tropicalis* and *X. laevis*, whereas only 23 exons were successfully sequenced in all taxa (Figure 4). To identify potential contamination or incongruence due to gene duplication/loss, we report all clades with a bootstrap proportion ≥0.7 across gene trees (Table S1).

In concatenated analyses, bootstrap proportions for almost all nodes were 1.0, and Bayesian concordance factors were over 0.75 (Figure 4). The only exception was the node that represents the common ancestor among *X. tropicalis*, *X. laevis*, and *Hymenochirus curtipes*: the bootstrap proportion was 0.61 for the unpartitioned analysis, 0.75 for the analysis partitioned by exon, and, using the data set with no missing data, 0.86 for the unpartitioned analysis and 0.92 when partitioned by exon. For this node, the Bayesian concordance factors based on 23 exons sampled for all 13 taxa was 0.817. *Rhinophrynus dorsalis* is widely accepted on the basis of morphological and molecular data as sister to Pipidae [47], [48], [49], [50], [51], and gene trees that place this taxon elsewhere in the tree could indicate lack of orthology or systematic error affecting phylogenetic reconstruction for that exon. After excluding exons that failed to infer *Rhinophrynus dorsalis* as sister to pipid frogs, a maximum-likelihood analysis of the remaining exons (n = 718) returned a bootstrap proportion of 1.0 supporting *X. tropicalis+X. laevis+H. curtipes*.

### Orthology Assessment

Targets were selected only if they were single-copy in the *Xenopus* genome, but they may not necessarily be single-copy across all frogs. Gene duplications interfere with phylogenetic reconstruction when different copies are independently lost (or fail to be sequenced) in descendants, such that paralogs, rather than orthologs, are analyzed. Orthology is difficult to assess in species for which there is no complete genome. Alignments of genes that result in strongly supported and conflicting topologies, compared to analyses of the concatenated dataset, may be candidates for containing paralogous sequences [6]. We identified bipartitions that were strongly supported and yet differed from the concatenated tree (in 32 gene trees; Table S1). Those gene trees that do have strong, incongruent signal with our concatenated tree may have been inferred from sequences that are paralogous rather than orthologous–or these incongruences may represent hybridization, incomplete lineage sorting, or systematic error. Because the underlying cause of incongruence was uncertain, we still included these exons in our concatenated analyses, except when explicitly testing their possible effect on relationships within Pipidae.

## Discussion

We demonstrated that targeted enrichment is an efficient method for generating phylogenomic data sets for distantly-related species. A single reference genome can be used to design probes for targeted enrichment of loci, even if the reference genome is distantly related and no other information regarding phylogenetic utility of targeted loci is available. Our resulting phylogeny is well supported and is congruent with published phylogenies based on morphological characters and other molecular sequence data (e.g., [47], [48], [49], [50]).

We found the main limitation to the microarray-based enrichment protocol compared to the solution-based protocol was its sensitivity to the presence during hybridization of species-specific, multi-copy DNA (*C_0_t*-1 DNA), which requires sufficient tissue to produce. For many samples, we had small amounts of tissue (less than 1 g), and would not have been able to make *C_0_t*-1 DNA for all of them. The solution-based protocol was successful at generating large amounts of data across frog species separated by millions of years. For example, between the reference species *Xenopus tropicalis* and its congener *X. laevis*, which diverged ∼65 mya (Figure 1), 711 target exons were successfully sequenced at 15× coverage with a minimum uniformity of 0.4 (Table 3). As expected, enrichment was greater for regions of the genome that appear to be more evolutionarily conserved (Table 4). Although targeted enrichment was effective over deep evolutionary time, as divergence time between the reference species and the species tested grows, enrichment (and thus the number of target exons sequenced) decreases (Figure 3). We found enrichment performed most efficiently when the target and reference species diverged between 0–200 mya; at this hierarchical level about 90 exons were sequenced across all taxa in the clade. However, only 23 exons were sequenced in all taxa across frogs, representing a divergence of ∼245 mya. This is driven in part by a drop-off in overall enrichment for each species (Figure 3), but also because at deeper evolutionary times, successful enrichment varies greatly. For example, whereas 89 exons were successfully enriched in *Hyla chrysoscelis*, only 34 of these were shared with another neobatrachian, *Bufo nebulifer* (Figure 4). Our experiment was not designed to distinguish between enrichment variation due to differences in DNA quality, in nonstationary gene evolution within taxa, or *in vitro* stochastic effects on hybridization to probes or sequencing reads.

Substitution rates in neobatrachians have been estimated to be elevated relative to other frogs [50], [52]. We observe reduced enrichment in the neobatrachians sampled for this study, *Bufo* (n = 58), *Hyla* (n = 89), and *Heleophryne* (n = 55), compared to other species, even when they share the same most recent common ancestor with *Xenopus* (i.e., *Scaphiopus*, n = 159) or older (such as *Discoglossus*, n = 184). In contrast to other studies, our maximum-likelihood estimate does not have statistically significantly longer branches leading to the neobatrachians compared to other frogs, suggesting that enriched exons do not have enhanced substitution rates (Table 4). If faster substitution rates have led to greater sequence divergence in neobatrachians across most of the genome, probes developed based on *Xenopus* may be binding only those homologous sequences that are more evolutionarily conserved, and failing to enrich many other exons.

### The Frog Tree of Life

We analyzed an unprecedented number of variable loci to generate a phylogeny of Anura (Figure 4). Our phylogenetic tree had high statistical support, regardless of the partitioning scheme or proportion of missing data: bootstrap proportions were 1.0 for all nodes with the exception of the node that places *Hymenochirus* as sister to *Xenopus.* In contrast to other studies in which bootstrap proportions increase as incomplete data are added to the alignment (reviewed in [53]), our study found that support for the placement of *Hymenochirus* actually *decreased* as more sequence (and gaps) were added: the bootstrap proportion based on analyzing the matrix with no missing data was 0.92, while in the larger matrix (with gaps) it fell to 0.75.

Rooting Pipidae using analysis of DNA sequence data is a difficult problem. In a study across Anura, using mitogenomes and nine nuclear protein-coding gene sequences, Irisarri et al. [49] found an identical topology to Figure 1, and similar bootstrap support for *Hymenochirus*+*Xenopus* (0.73). In contrast, studies that sampled within Pipidae have concluded that *Hymenochirus* and *Pipa* are sister taxa [51], [54]. Bewick et al. [51] described support for alternate rooting across gene trees, with approximately equal numbers of gene trees supporting each of three possible rooted pipid phylogenies: *Hymenochirus*+*Xenopus/Silurana*, *Hymenochirus*+*Pipa*, and *Pipa*+*Xenopus/Silurana*, although they argued that the largest posterior distribution of gene trees support *Hymenochirus*+*Pipa*. In this study, bootstrap support for *Hymenochirus*+*Xenopus* varies across maximum-likelihood estimates for individual target exons with an average bootstrap proportion of 0.53, compared to only 0.14 for *Hymenochirus*+*Pipa*. However, only seven gene trees had a bootstrap proportion ≥0.7 for *Hymenochirus*+*Xenopus*, compared to only two for *Hymenochirus*+*Pipa* (Table S1). Sample-wide Bayesian concordance factors, using only those 23 exons sampled across frogs, also demonstrate higher support for *Hymenochirus*+*Xenopus*, 0.817, but only 0.073 for *Hymenochirus*+*Pipa*. *Rhinophrynus dorsalis* is the sister taxon to the pipid frogs, and thus determines the placement of the pipid root [47], [49], [51]. Gene trees that place *R. dorsalis* as sister to a non-pipid taxon with high support are unexpected, and either gene duplication and loss or experimental error may have caused paralogs rather than orthologs to be sequenced. When we exclude exons that place *R. dorsalis* sister to some other taxon with a proportion ≥0.7, the maximum-likelihood estimate of the concatenated alignment recovers *Xenopus+Hymenochirus* with a bootstrap proportion of 1.0. Conflicting estimates among data sets can also be caused by underlying biological processes such as incomplete lineage sorting (i.e., deep coalescence) or hybridization between lineages shortly following speciation. Thus we would recommend increased taxon sampling within Pipidae, coupled with analyses designed to test alternative hypotheses for the cause of incongruence.

### Comparison with Other Methods

For phylogenetic analysis, data matrices of orthologous genes with relatively complete data are ideal. Transcriptomes are increasingly being used as data for phylogenetic analyses [16], [17], [18]. Sequencing transcriptomes enriches samples for exon regions, but these approaches require fresh or fresh-frozen tissue samples from which mRNA can be reliably extracted, and will only produce gene sequences from genes that are expressed at the time and in the tissue of extraction. As a result, transcriptome analyses tend to produce aligned matrices with many missing characters (gaps). Across all frogs, our alignment is similarly missing large numbers of characters. For example, a transcriptome analysis in a clade of mosquitoes that diverged about 145–200 mya [55] resulted in an alignment with comparable size, but with a greater percentage of missing data, when compared to the ∼195 my-old clade containing *Rhinophrynus dorsalis* and the pipid frogs (∼389,000 bp with 51% missing data in their study [16] compared to our ∼200,000 bp with 30% missing data). The main advantage to our approach over transcriptome sequencing is that fresh tissue is not required for enrichment, and that potentially phylogenetically informative, non-coding flanking regions could also be sequenced as a consequence of the enrichment process.

Probes based on single genomes have been successfully applied to enrich genes in taxa that share a common ancestor as long ago as 33 mya [28]. At deeper time scales, multiple genomes have been used to identify conserved regions across species for enrichment [25], [26]. In our study, we tested whether probes based on a single genome could perform well across a wide range of divergence times. Comparing results across studies is complicated by the difference in next-generation sequencing technologies used, which impacts read length and quality, and thus contig assembly. However, studies that have used conserved regions across multiple genomes appear to have somewhat similar results. For example, Lemmon et al. successfully enriched 69–512 loci for taxa that spanned 400 my; 69–258 loci were shared among taxa that have a common ancestor ∼250 mya (when assembly was conservative; [26]). A key component to assessing probe design, however, is also assessing the amount of phylogenetically-informative variation within contigs [27], which will include both the target region and flanking sequences. Our range of sequence variation among taxa that shared a common ancestor <65 mya (i.e., between *Xenopus* species; Table 4) appears similar to results of Faircloth et al., who reported values between 0 and 0.15 and enrichment success of 75% [25]. Testing multiple probe designs within the same probe pool on taxa that range in divergence times would allow for direct comparison of phylogenetically-informative variation.

### Conclusions

We have developed an approach for effectively generating data appropriate for phylogenetic analysis across deeply-diverged taxa. This technique simultaneously sequences hundreds of loci from genomic DNA obtained from small amounts of tissue, museum specimens, or small organisms, and only requires a single reference genome or transcriptome within the clade of interest. The overall approach is expected to be useful for organisms that not only belong to the same species, but which share a common ancestor millions of years in the past. Expansion of these protocols to develop large-scale datasets that are well-sampled taxonomically is a next step in the path towards building an accurate Tree of Life.

## Supporting Information

Table S1 **Number of maximum likelihood estimates of gene trees with bootstrap support ≥70 for described bipartitions.**
(DOCX)
