# Supplementary material for: Targeted Enrichment: Maximizing Orthologous Gene Comparisons across Deep Evolutionary Time
Source: PLoS One. 2013 Jul 2;8(7):e67908. doi: 10.1371/journal.pone.0067908 (PMC3699465; doi:10.1371/journal.pone.0067908)
Supplement: Table S1 — Number of maximum likelihood estimates of gene trees with bootstrap support ≥70 for described bipartitions. (DOCX) [file pone.0067908.s001.docx]

| **Bipartitions inconsistent with concatenated MLE** | **# gene trees** |
| --- | --- |
| *Hymenochirus curtipes* + *Pipa pipa* | 2 |
| *Xenopus tropicalis* + *Hymenochirus curtipes* (no *Pipa pipa* in alignment) | 2 |
| *Hymenochirus curtipes* + *Hyla chrysoscelis* | 1 |
| *Xenopus laevis* + *Pipa pipa* | 1 |
| *Xenopus tropicalis* + *Pipa pipa* | 1 |
| *Xenopus tropicalis* + *X. laevis* + *Pipa pipa* | 3 |
| *Xenopus laevis* + *Hymenochirus curtipes* | 6 |
| *Rhinophrynus dorsalis* + *Discoglossus pictus* | 4 |
| *Rhinophrynus dorsalis* + *Scaphiopus hurterii* | 3 |
| *Rhinophrynus dorsalis* + *Scaphiopus hurterii* + *Hyla chrysoscelis* | 1 |
| *Rhinophrynus dorsalis* + *Discoglossus pictus* + *Ascaphus montanus* | 1 |
| *Rhinophrynus dorsalis* + *Discoglossus pictus* + *Bombina variegata* | 1 |
| *Rhinophrynus dorsalis* + *Ascaphus montanus* | 1 |
| *Rhinophrynus dorsalis* + *Bombina variegata* | 2 |
| *Rhinophrynus dorsalis* + *Bombina variegata* + *Discoglossus pictus* | 1 |
| *Scaphiopus hurterii* + *Ascaphus montanus* | 1 |
| *Bufo nebulifer* + *Hyla chrysoscelis* + *Heleophryne purcelli* + *Discoglossus pictus* | 1 |
| *Discoglossus pictus* + *Ascaphus montanus* | 2 |
| *Ascaphus montanus* + *Hyla chrysoscelis* | 1 |
| *Bombina variegata* + *Discoglossus pictus* + *Ascaphus montanus* | 1 |
| *Bombina variegata* + *Ascaphus montanus* | 1 |
| **Bipartitions consistent with concatenated MLE (given taxon sampling for that exon)** |  |
| *Xenopus tropicalis* + *Xenopus laevis* | 90 |
| *Xenopus tropicalis* + *Xenopus laevis* + *Hymenochirus curtipes* | 7 |
| *Xenopus tropicalis* + *Xenopus laevis* + *Hymenochirus curtipes* (no *Pipa pipa*) | 40 |
| *Xenopus tropicalis* + *Xenopus laevis* + *Pipa pipa* (no *Hymenochirus curtipes*) | 3 |
| *Xenopus tropicalis* + *Xenopus laevis* + *Hymenochirus curtipes* + *Pipa pipa* | 38 |
| *Xenopus tropicalis* + *Xenopus laevis* + *Hymenochirus curtipes* + *Pipa pipa* + *Rhinophrynus dorsalis* | 3 |
| *Xenopus tropicalis* + *Xenopus laevis* + *Hymenochirus curtipes* + *Rhinophrynus dorsalis* (no *P. pipa*) | 2 |
| *Bufo nebulifer* + *Hyla chrysoscelis* | 26 |
| *Bufo nebulifer* + Hyla chrysoscelis + *Heleophryne purcelli* | 12 |
| *Bufo nebulifer* + *Hyla chrysoscelis* + *Heleophryne purcelli* + *Scaphiopus hurterii* | 2 |
| *Scaphiopus hurterii* + *Hyla chrysoscelis* | 4 |
| *Ascaphus montanus* + *Leiopelma hochstetteri* | 14 |
| *Discoglossus pictus* + *Bombina variegata* | 18 |
| *Discoglossus pictus* + *Leiopelma hochstetteri* | 1 |
| *Discoglossus pictus* + *Ascaphus montanus* | 1 |
| *Discoglossus pictus* + *Ascaphus montanus* + *Leiopelma hochstetteri* | 1 |
| *Bombina variegata* + *Discoglossus pictus* + *Ascaphus montanus* | 1 |
| *Bombina variegata* + *Discoglossus pictus* + *Ascaphus montanus* + *Leiopelma hochstetteri* | 1 |
| *Bombina variegata* + *Ascaphus montanus* | 1 |
